# Pilot study for risk assessment of aspiration pneumonia based on oral bacteria levels and serum biomarkers

**DOI:** 10.1186/s12879-019-4327-2

**Published:** 2019-09-02

**Authors:** Tomotaka Nishizawa, Yuichi Niikura, Keiichi Akasaka, Masato Watanabe, Daisuke Kurai, Masako Amano, Haruyuki Ishii, Hidekazu Matsushima, Naomi Yamashita, Hajime Takizawa

**Affiliations:** 1 Department of Respiratory Medicine, Japanese Red Cross Society Saitama Hospital, 1-5 Shintoshin Chuo-ku Saitama-shi, Saitama, 330-8553 Japan; 2 Department of Pharmacotherapy, Research Institute of Pharmaceutical Sciences Musashino University, Tokyo, Japan; 3 Department of Respiratory Medicine, Graduate School of Medicine, Kyorin University, Tokyo, Japan; 4 Department of General Medicine, Kyorin University, Tokyo, Japan

**Keywords:** Aspiration pneumonia, Bacterial count, Oral hygiene

## Abstract

**Background:**

Aspiration pneumonia is a serious problem among elderly patients; it is caused by many risk factors including dysphagia, poor oral hygiene, malnutrition, and sedative medications. The aim of this study was to define a convenient procedure to objectively evaluate the risk of aspiration pneumonia in the clinical setting.

**Methods:**

This prospective study included an aspiration pneumonia (AP) group, a community-acquired pneumonia (CAP) group, and a control (Con) group (patients hospitalized for lung cancer chemotherapy). We used the Oral Health Assessment Tool (OHAT), which assesses oral hygiene, and evaluated performance status, body mass index, serum albumin levels, substance *P* values in plasma, and oral bacterial counts.

**Results:**

The oral health as assessed by the OHAT of the aspiration pneumonia group was significantly impaired compared with that of the CAP group and the control (5.13 ± 0.18, 4.40 ± 0.26, 3.90 ± 0.22, respectively; *p* < 0.05). The oral bacterial count in the aspiration pneumonia group (7.20 ± 0.11) was significantly higher than that in the CAP group (6.89 ± 0.12), consistent with the OHAT scores. Oral bacterial count was significantly reduced by oral care.

**Conclusions:**

OHAT and oral bacterial counts can be a tool to assess the requirement of taking oral care and other preventive procedures in patients at high risk of aspiration pneumonia.

## Background

Pneumonia is one of the most frequent causes of death among the elderly [1]. Aspiration pneumonia can develop after the inhalation of pathogenic bacteria into the lower respiratory tract, and can present as ventilator-associated pneumonia, hospital-acquired pneumonia, or community-acquired pneumonia. Patients with swallowing dysfunction, gastroesophageal dysfunction, or mechanical obstruction are particularly susceptible to aspiration pneumonia [2, 3]. Aspiration pneumonia accounts for 5–53.2% of hospitalized adult pneumonia cases; the incidence tends to be higher in Japan [2, 4, 5]. In fact, more than 70% of pneumonia cases in the elderly have been reported to be aspiration pneumonia in Japan [6]. Moreover, the mortality of patients with aspiration pneumonia is higher than that for other types of pneumonia [7].

One important risk factor for aspiration pneumonia is oral health [8]. Recently, an oral bacterial count-measuring device has been developed in the dental field to check the condition of the oral cavity [9]. Using this device, oral bacteria counts in the elderly have been shown to be a risk factor for aspiration pneumonia [10]. Moreover, oral care that reduces bacteria has been reported to suppress the onset of aspiration pneumonia [11–13]. In particular, oral care by dentist or oral hygienists has proven to help prevent aspiration pneumonia [14, 15]. However, in many clinical situations such as nursing care in residential care facilities, it is difficult for the experts to perform oral care; therefore, it is important to standardize oral care procedures and to objectively assess the quality of oral care.

Another risk factor for aspiration pneumonia is swallowing dysfunction [2, 5]. In elderly people, microaspiration frequently occurs daily because the cough reflex is attenuated during deglutition [16]. This reflex abnormality is related to a decrease in substance P, which regulates the cough reflex [17, 18]. Substance P is a peptide that is synthesized in response to the stimulation of dopamine in the brain and is secreted into the pharynx and trachea [19, 20]. In the elderly and patients with cerebrovascular disorders, cerebral blood flow is decreased, which leads to reduction in substance P [21]. Administration of angiotensin-converting enzyme inhibitors (ACEIs), dopamine synthesis-promoting agents, or cerebral blood flow-improving drugs has been shown to ameliorate and prevent aspiration pneumonia [22–24]. However, the usefulness of substance P as a risk factor for aspiration pneumonia has not been confirmed.

In this study, we compared oral health and serum markers in patients with aspiration pneumonia to those in patients with community acquired pneumonia. We employed the Oral Health Assessment Tool (OHAT), which was developed in the dental field, to assess problems in the oral cavity [25]. In addition, we monitored oral care, assisted by the nurse if necessary, according to an established protocol and checked bacterial counts by using a bacteria count-measuring instrument to evaluate oral care objectively.

## Methods

### Patients

This study was performed as a prospective cohort study of hospitalized patients at the Japanese Red Cross Society Saitama Hospital in Saitama City, Saitama Prefecture, Japan. We examined 62 elderly adults, aged older than 50 years, who were admitted to the hospital and agreed to participate in this study from June 2017 to May 2018. One pulmonologist examined all of the patients and confirmed the diagnosis of pneumonia with other pulmonologists. The imaging diagnosis of pneumonia was made jointly by a pulmonologist and a radiologist. We evaluated the number of bacteria in the oral cavity, oral hygiene, and serum biomarkers among three groups: aspiration pneumonia (AP), community-acquired pneumonia (CAP), and control (Con).

In this study, patients were assigned to the AP group if they had risk factors for the development of pneumonia after aspiration (cerebrovascular disorder, swallowing disorder, chronic neurological disease, dementia, etc.) and gravity-dependent lung segments on chest CT imaging (superior lower-lobe or posterior upper-lobe segments, if the patient was in a spine position during the event; or basal segments of the lower lobe, if the patient was upright) [26, 27]. We excluded patients with HAP (hospital-acquired pneumonia) or VAP (ventilator-associated pneumonia).

In addition, patients were excluded if we suspected acid-fast bacterial infection or chronic respiratory tract infection associated with structural destruction of the lungs, such as sequelae of tuberculosis and bronchiectasis. Patients were assigned to the CAP group according to IDSA (Infectious Diseases Society of America)/ATS (American Thoracic Society) consensus guidelines on the management of community-acquired pneumonia in adults; those with overt aspiration or HAP, VAP, or HCAP (health care-associated pneumonia) were excluded [28, 29]. Patients enrolled in the CAP group were also disease free (i.e., no cerebrovascular disorder, Parkinson’s disease, dementia, etc.), which could have influenced the risk of aspiration.

We confirmed that no antibiotics were given prior to hospitalization. We did not use invasive blood pressure monitoring or tube feeding. We recruited non-ICU (intensive care unit) setting patients. When we evaluated the recruited patients by using CURB-65, none were rated at 3–5 points, which would have indicated that they should be in the ICU setting. In the AP (aspiration pneumonia) group, 14 patients were at 1 point and 8 patients, at 2 points, whereas in the CAP (community-acquired pneumonia) group, 10 patients were at 1 point and 10 patients, at 2 points. Therefore, the patients did not receive medications such as sedative drugs, painkillers, muscle relaxants, benzodiazepines or opioids. In addition, patients who used ACEIs were excluded. We also confirmed that there were no illicit drug users in all groups.

We included patients who were hospitalized for lung cancer chemotherapy as the Con group; we excluded patients who had metastatic brain tumors. For patients admitted for chemotherapy, blood tests and OHAT were evaluated prior to induction of chemotherapy at admission. There was no bone marrow suppression. We confirmed that the Con patients were in the stable phase. Performance status (PS) was evaluated by using the ECOG classification (0–4).

### Data collection

To collect the oral bacterial count sample, we scratched the center of the tongue back using a sterile cotton swab attached to a constant pressure specimen collection device according to the manufacturer’s instruction (PHC Ltd., Tokyo, Japan) [30]. For the analysis, we used a bacterial count-measuring instrument (bacteria counter PHC Ltd., Tokyo, Japan) [9]. This instrument collects bacteria in a liquid on an electrode, measures the change in impedance, and converts it into a bacterial concentration (cfu /ml) in 1 ml of sample.

To evaluate oral health, we used the OHAT. The reliability and validity of this tool was assessed in 485 residents in residential care facilities and shown to significantly correlate with other oral hygiene evaluations [25]. The tool evaluates visually eight items: lips, tongue, gums/tissues, saliva, natural teeth, dentures, oral cleanliness, and dental pain. Each item is divided into three stages: 0 = healthy, 1 = oral changes and 2 = unhealthy. OHAT score mean the higher the number the worse the oral health.

During hospitalization, we provided oral care, following an established protocol with some modification [31]. For patients who were not self-supporting, nurses carried out mouth care using a toothbrush and sponge brush.

For serum markers, albumin (Alb) was measured as a factor related to nutrition. C-reactive protein (CRP), CRP/Alb, and white blood cell (WBC) were also measured as a serological evaluation of the inflammatory response and pneumonia. To measure substance P, we collected the blood of patients using EDTA-Na plus aprotinin tubes and immediately centrifuged them at 0 °C, 160×*g* for 15 min. Then the plasma components were cryopreserved at − 80 °C until measurements were obtained. For sample purification, an equal amount of 1% trifluoroacetic acid (TFA) was added to the sample, which was then centrifuged at 4 °C, 17000×*g* for 15 min, and the supernatant was collected. Next, 1 ml of acetonitrile and 15 ml of 1% TFA were added to a 200 mg Strata C18 Sep column (Phenomenex, Torrance, California, USA). The supernatant was then added to the Sep column and washed with 15 ml of 1% TFA. After that, the sample was eluted and extracted into a plastic tube while slowly adding 3 ml of a solution of acetonitrile:1% TFA (60:40). The extract was dried using a centrifuge and stored at − 20 °C. The substance P concentration was measured by using an Enzyme-Linked ImmunoSorbent Assay (ELISA) (Enzo Life Science Inc., Farmingdale, New York, USA). The extracted sample was dissolved in 100 μl of assay buffer. The sample (50 μl), reference substance P (50 μl), and anti-substance P antibody (50 μl) were added to each well. The reaction was carried out at room temperature for 2 h with horizontal shaking at 350 rpm on a microplate shaker. Each well was then washed three times with wash buffer, p-nitrophenyl phosphate substrate was added, and the reaction was carried out for 1 h at room temperature. Finally, trisodium phosphate was added as stop solution, and the absorbance was measured immediately at a wavelength of 405 nm by using a BioRadmodel 680 microplate reader (Bio-Rad, US) and corrected at 570 nm [32].

### Statistical analysis and data management

Data were analyzed using JMP pro v.13.0 software (SAS Institute Inc., Cary, North Carolina, USA). An analysis of variance was performed to compare the three groups. The Wilcoxon or Kruskal-Wallis test was used to assess the relationship between groups. Receiver operating characteristic (ROC) curves were used to evaluate the sensitivity and specificity of the AP group vs the Con group (OHAT score), the Con group vs the CP group (OHAT score), and the CP group vs the AP group (OHAT score). Cut-off values are reported with the area under the curve (AUC) being given with its 95% confidence interval (CI). The cut-off values were calculated by using the Youden index.

The paired *t*-test was used to compare the effect of oral care assessed by bacterial count between before and after care. All *p*-values were two-tailed and considered significant for *p* < 0.05. GraphPad Prism 7.0 (GraphPad Software, San Diego, California, USA) was used for graph plotting.

### Ethics, consent, and permissions

Written informed consent was obtained from all subjects or their surrogates. The ethics committee of the Japanese Red Cross Society Saitama Hospital and Musashino University, ID n.17-c approved this study and the informed consent procedure (No. 1 and No. 62, respectively). Dr. Keiichi Akasaka, Japanese Red Cross Saitama Hospital, anonymized the specimen samples. He also managed all personal and anonymized information. We sent the anonymized, numbered samples to Musashino University. All phases of the study were carried out in accordance with the principles expressed in the WMA Declaration of Helsinki.

## Results

The characteristics of the three patient groups are described in Table 1. Regarding age, there was no significant difference between the AP and the CAP group; however, the Con group was significantly younger than the AP and CAP groups. The percentage of men in the AP group was 50%, whereas it was 80% in the CAP group, and 70% in the Con group; however, the difference was not significant. In the AP group, patients had risk factors for aspiration such as cerebrovascular disorder, swallowing disorder, or chronic neurological disorder (Table 1); such risk factors were absent from both the CAP and Con groups.

**Table 1:** Baseline characteristics

	AP	CAP	Con	
	(*n* = 22)	(*n* = 20)	(*n* = 20)	
Age, mean±standard deviation (years)	84.7 ± 6.1	81.3 ± 7.4	73.6 ± 8.5	AP vs CAP NS
				AP vs Con and
				CAP vs Con *p* <0.001
Male, number (%)	11 (50)	16 (80)	14 (70)	
Underling Disease (%)				
cerebrovascular disease	13 (59.0)	0	0	
swallowing disorder	9 (27.3)	0	0	
chronic neurological disease	2 (9.1)	0	0	
dementia	8 (36.4)	0	0	
diabetes mellitus	3 (13.6)	3 (15.0)	5 (25.0)	
COPD	3 (13.6)	7 (35.0)	7 (35.0)	

Regarding diabetes mellitus, which is a risk factor for infection, the proportion in the AP group (13.6%) and the CAP group (15.0%) was not significantly higher than that in the Con group (25.0%). Chronic obstructive pulmonary disease (COPD), which is as a risk factor of aspiration, was not significantly higher in the AP group (13.6%) compared with the CAP and Con group.

We compared PS, body mass index (BMI), and blood test results among the three groups (Table 2). The PS of the AP group (2.7 ± 1.0) was significantly worse than that of the CAP group (1.6 ± 0.6). In addition, the PS of the CAP group was significantly worse than that of the control group (1.1 ± 0.4). We also observed significant differences among the three groups with respect to BMI, albumin, CRP, and WBC (AP and CAP vs Con).

**Table 2:** Comparison of physical characteristics and serum markers of patients

	AP	CAP	Con	
	(*n* = 22)	(*n* = 20)	(*n* = 20)	
PS	2.7 ± 1.0	1.6 ± 0.6	1.1 ± 0.4	AP vs CAP,
				AP vs Con and
				CAP vs Con *p* <0.0001
BMI (kg/m^2^ )	17.6 ± 4.1	19.8 ± 4.3	23.0 ± 3.5	AP vs CAP NS
				AP vs Con and
				CAP vs Con *p* =0.0005
Alb (g/dL)	3.14 ± 0.67	3.19 ± 0.57	3.62 ± 0.50	AP vs CAP NS
				AP vs Con and
				CAP vs Con *p* =0.00013
CRP (mg/dL)	9.52 ± 8.49	12.63 ± 7.76	2.28 ± 5.91	AP vs CAP NS
				AP vs Con and
				CAP vs Con *p* <0.0001
CRP/Alb	3.64 ± 3.88	4.3 ± 3.15	0.85 ± 2.55	AP vs CAP NS
				AP vs Con and
				CAP vs Con *p* <0.0001
WBC (/μL)	10430.9 ± 4990.9	11809.5± 3538.8	7299.5 ± 3810.7	AP vs CAP NS
				AP vs Con and
				CAP vs Con *p* =0.001

Data are expressed as mean ± standard deviation

**Fig. 1:**
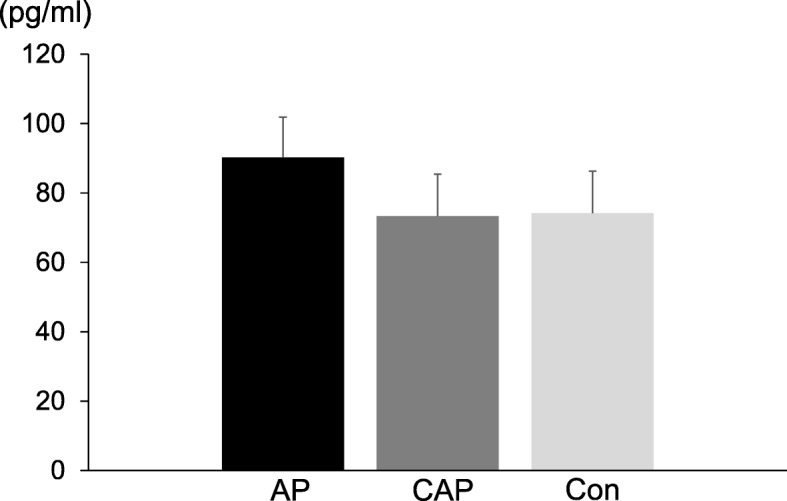
Level of substance P. Abbreviations: AP, aspiration pneumonia group; Con, control group; CAP, community-acquired pneumonia group. The blood of patients was collected using EDTA-Na plus aprotinin tubes. After purification, as described in the Methods section, the level of substance P in the plasma was measured by using specific ELISA kits

Next, we examined whether substance P could serve as a marker for aspiration pneumonia. We did not find any difference in plasma substance P levels among the three groups, as shown in Fig. 1 (AP group, 90.2 ± 11.5; CAP group 73.2 ± 12.1; and Con group 74.1 ± 12.1).

**Fig. 2:**
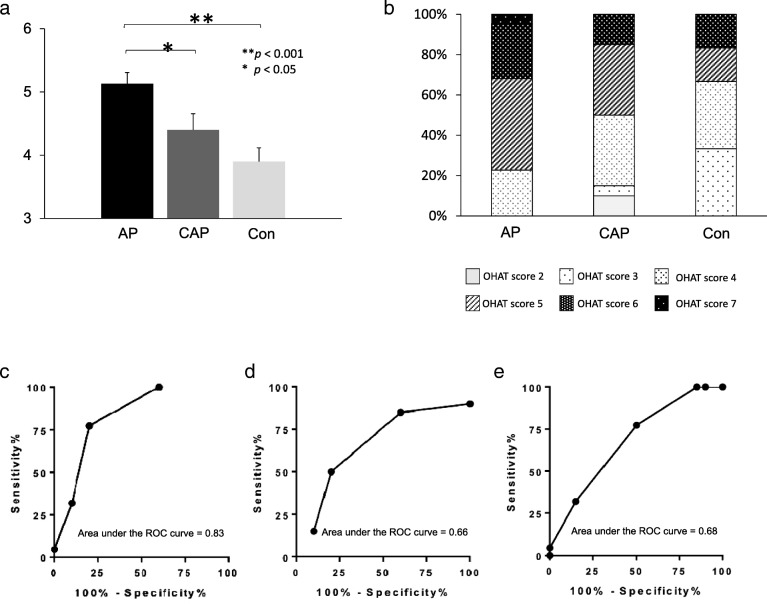
OHAT. **a** OHAT score, **b** OHAT score ratios, **c** ROC curve of the AP group vs the Con group (OHAT), **d** ROC curve of the Con group vs the CAP group (OHAT), **e** ROC curve of the CAP group vs the AP group (OHAT). **a** Oral hygiene was assessed by using the OHAT score system. **b** Each OHAT score ratio is shown. In a panel lavel, each fill pattern is displayed separately for each OHAT score. **c** The area under the ROC curve is shown. **d** The area under the ROC curve is shown. **e** The area under the ROC curve is shown. Abbreviations: AP, aspiration pneumonia group; Con, control group; CAP, community-acquired pneumonia group; OHAT; oral health assessment tool; ROC, Receiver Operating Characteristic

At the time of admission, we checked the oral health of the patients by using the OHAT. The OHAT score was 5.13 ± 0.178 in the AP group, 4.40 ± 0.255 in the CAP group, and 3.90 ± 0.216 in the Con group (Fig. 2a, AP vs CAP *p* < 0.05; AP vs Con *p* < 0.001). Next, we compared the distribution of the OHAT score in each group (Fig. 2b). An OHAT score of 7 was found only in the AP group. Scores of 6 and 5 predominated in the AP group, which had the lowest percentage of score 4 among the groups. Scores of 3 and 2 were not observed in the AP group. These data suggest that oral health was worst in the AP group.

In ROC analysis, OHAT score differentiated the AP group from the Con group with an AUC of 0.83. Calculating Youden index, we optimized the cut-off value to be 4.5, which diagnosed the AP group with a sensitivity of 77.3 and a specificity of 80 (Fig. 2c). Similarly, OHAT score differentiated the CAP group from the Con group with an AUC of 0.66, where the optimized cut-off value was also 4.5 being diagnostic of the CAP group with a sensitivity of 50 and a specificity of 80 (Fig. 2d). Odds ratios (95% CI) that high OHAT score (> 4.5) predicted the AP group and the CAP group were 13.6 (56.34 to 94.27%) and 4 (56.34 to 94.27%), respectively. Further, OHAT score also differentiated the AP group from the CAP group with an AUC of 0.68, where the optimized cut-off value of 5.5 diagnosed the AP group with a sensitivity of 31.8 and specificity of 85 (Fig. 2e). These indicate that high OHAT score is diagnostic of aspiration pneumonia.

**Fig. 3:**
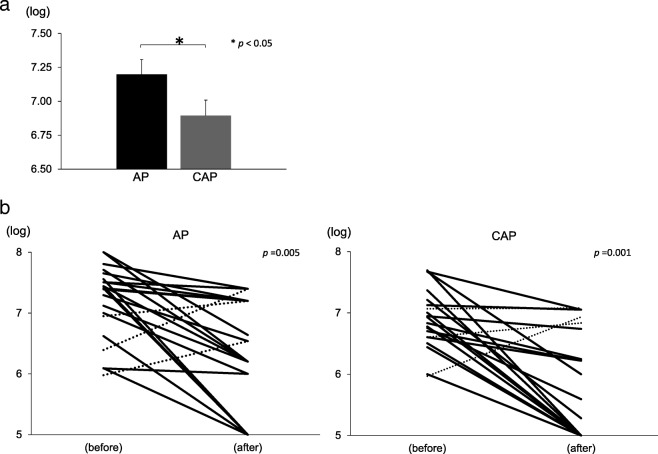
Oral bacterial counts. **a** Oral bacterial counts, **b** Effect of oral care as assessed by bacterial count. **a** Oral bacteria levels at the time of admission were analyzed using a bacteria count-measuring instrument, which is a dielectrophoretic impedance measuring system. The bacterial concentration (cfu/ml) in 1 ml of a sample is shown in a logarithmic scale. **b** Oral care was performed by nursing staff. In comparison before and after oral care, the solid lines indicate that oral bacterial counts have decreased, and the dotted lines indicate that oral bacterial counts have increased. Abbreviations: AP, aspiration pneumonia; Con, control; CAP, community-acquired pneumonia

We also assessed the number of oral bacteria at the time of admission by using a bacteria count-measuring instrument, which is dielectrophoretic impedance measuring system that exhibits a good correlation to the actual number of bacteria in the oral cavity [33]. As shown in Fig. 3, the oral bacterial count (logarithm) in the AP group (7.20 ± 0.110) was significantly greater than that in the CAP group (6.89 ± 0.115), which is consistent with the OHAT scores. Next, we compared bacteria counts before and after oral care in the AP and the CAP group. We found that bacterial count was successfully decreased by oral care during hospitalization in both the AP and CAP groups (Fig. 3a and b, respectively), suggesting that oral care for hospitalized patients successfully improves their oral health.

## Discussion

In this study, we showed that the OHAT and oral bacterial count are useful for assessing the risk of aspiration pneumonia in the aspect of oral health. The oral assessment tool ROAG (Revised Oral Assessment Guide) has been widely used in the field of nursing care [34]. OHAT, however, is more precise than ROAG in terms of assessing the state of cleaning in the oral cavity and because the remaining teeth and dentures are evaluated as separate items. Therefore, we believe that the OHAT is superior to the ROAG for evaluations related to the oral phase of swallowing [35]. Whereas the OHAT is highly valued in the field of swallowing rehabilitation, to our knowledge, our study is the first to use this tool to assess oral health as a risk factor for pneumonia. We found the OHAT to be useful for distinguishing between patients with aspiration pneumonia and control patients, and its diagnostic value to be very high.

In addition, PS and nutritional condition were also risk factors for aspiration pneumonia, consistent with previous reports [36]. BMI and serum albumin concentration in the AP and CAP groups were significantly lower than those in the Con group, suggesting that improving nutrition status may have a beneficial effect on pneumonia.

Kikuchi et al. studied inapparent aspiration by using indium chloride as a tracer and reported a significantly higher percentage of inapparent aspiration among patients with a history of acute pneumonia compared with control group (71% vs 10%, respectively) [16]. Together with our results, these data suggest that we should provide more intensive oral care to acute pneumonia patients to prevent aspiration pneumonia [16]. There are nearly 1 × 10^8^ bacteria in every milliliter of saliva with over 700 species represented. They live in teeth, on the tongue, or in biofilms of the oral mucosa, but their numbers can be reduced by oral care [37–39]. In our study, nurses provided oral care and we compared its effect by examining oral bacteria counts before and after such care. We saw a significant improvement in patients in the AP and CAP groups, suggesting that oral care may reduce the incidence of aspiration pneumonia.

Substance P is a neurotransmitter that regulates the swallowing reflex and is reported to be decreased in aspiration pneumonia patients. Arai et al. reported that the concentration of serum substance P is significantly decreased in patients with swallowing disorder without hypertension. They also showed that ACEIs improved swallowing function in association with an increase in the serum level of substance P [22]. On the basis of these findings, we expected that the level of substance P would be a marker of swallowing disorders. However, our study showed no significant differences in the level of substance P concentration among the AP, CAP, and Con groups, in contrast to a previous report [19]. One reason for the discrepancy between these results might be the assay procedure. Another reason might be relatively low severity of our selected patients. We recruited non-ICU patients on the basis of CURB-65 scoring, in order to clearly distinguish AP and CAP. Therefore, patients with VAP, which is closely related to aspiration pneumonia, were excluded, as were patients with HAP. However, exact reason for the discrepancy remains to be cleared by further investigation.

### Limitations

There are several limitations in our study. One is the non-intervention study, which is not ideal to assess the effect of reducing the risk of aspiration pneumonia. Second is the small sample size. In order to improve our defect, we strictly follow diagnostic procedure and exclusion criteria by our respiratory department as shown in the methods. Still our finding needs to be evaluated with caution and validated by following studies.

## Conclusion

OHAT and oral bacterial counts could be tools for initiating oral care and other preventive measures in patients at high risk of aspiration pneumonia in the non-ICU setting. The effect of oral care can be easily assessed by using bacterial counts. Our findings may help to identify patients at higher risk of aspiration pneumonia and prompt appropriate instructions regarding dental issues and care after discharge from the hospital.

## Data Availability

Anonymized participant-level data are available upon request from the authors.

## Abbreviations

ACEIs: Angiotensin-converting enzyme inhibitors
Alb: Albumin
AP: Aspiration pneumonia
AUC: Area under the curve
BMI: Body mass index
CAP: Community-acquired pneumonia
CI: Confidential interval
COPD: Chronic obstructive pulmonary disease
CRP: C-reactive protein
CT: Computed tomography
HAP: Hospital-acquired pneumonia
HCAP: Healthcare-associated pneumonia
ICU: Intensive care unit
OHAT: Oral health assessment tool
PS: Performance status
ROAG: Revised oral assessment guide
ROC: Receiver operating characteristics
VAP: Ventilator associated pneumonia
WBC: White blood cell
